# Identification of Common Hub Genes in Human Dermal Fibroblasts Stimulated by Mechanical Stretch at Both the Early and Late Stages

**DOI:** 10.3389/fsurg.2022.846161

**Published:** 2022-04-18

**Authors:** Chen Dong, Wei Liu, Yu Zhang, Yajuan Song, Jing Du, Zhaosong Huang, Tong Wang, Zhou Yu, Xianjie Ma

**Affiliations:** Department of Plastic Surgery, Xijing Hospital, Fourth Military Medical University, Xi'an, China

**Keywords:** bioinformatics, genes, tissue expansion, dermis, fibroblasts, mechanical stretch

## Abstract

**Background:**

Mechanical stretch is vital for soft tissue regeneration and development and is utilized by plastic surgeons for tissue expansion. Identifying the common hub genes in human dermal fibroblasts (HDFs) stimulated by mechanical stretch at different stages will help elucidate the mechanisms involved and improve the efficiency of tissue expansion.

**Methods:**

A gene expression dataset (GSE58389) was downloaded from the Gene Expression Omnibus database. Differentially expressed genes (DEGs) in HDFs between cyclic mechanical stretching and static samples were identified at 5 and 24 h. Common DEGs overlapped in both the 5 h and 24 h groups. Gene Ontology (GO) and Kyoto Encyclopedia of Genes and Genomes (KEGG) pathway enrichment analyses were performed to determine the functions of the DEGs. Protein-protein interaction networks were constructed using the STRING database. The top 10 hub genes were selected using the plug-in Cytohubba within Cytoscape. The regulatory network of hub genes was predicted using NetworkAnalyst.

**Results:**

A total of 669 and 249 DEGs were identified at the early (5 h) and late stages (24 h), respectively. Of these, 152 were present at both stages and were designated as common DEGs. The top enriched GO terms were “regulation of autophagy” at the early stage, and “sterol biosynthetic processes” at the late stage. The top KEGG terms were “pyrimidine metabolism” and “synaptic vesicle cycle” at the early and late stages, respectively. Seven common DEGs [DEAD-box helicase 17 (*DDX17*), exocyst complex component 7 (*EXOC7*), CASK interacting protein 1 (*CASKIN1*), ribonucleoprotein PTB-binding 1 (*RAVER1*), late cornified envelope 1D (*LCE1D*), *LCE1C*, and polycystin 1, transient receptor potential channel interacting (*PKD1*)] and three common DEGs [5′-3′ exoribonuclease 2 (*XRN2*), T-complex protein 1 (*TCP1*), and syntaxin 3 (*STX3*)] were shown to be downregulated and upregulated hub genes, respectively. The GO terms of the common hub genes were “skin development” and “mRNA processing.” After constructing the regulatory network, hsa-mir-92a-3p, hsa-mir-193b-3p, RNA polymerase II subunit A (POLR2A), SMAD family member 5 (SMAD5), and MYC-associated zinc finger protein (MAZ) were predicted as potential targets in both stages.

**Conclusion:**

At the early stage, there were clear changes in gene expression related to DNA and chromatin alterations; at late stages, gene expression associated with cholesterol metabolism was increased. Common DEGs related to skin development, transcriptional regulation, and cytoskeleton rearrangement identified in both stages were found to be potential targets for promoting HDF growth and alignment under mechanical stretch.

## Introduction

Mechanical stretch is a force that is essential for the regeneration and development of skin and other soft tissues (1). Naturally, the growth and plasticity of skin and soft tissues can be observed when other structures grow within the body. For instance, embryo growth causes the abdominal skin to expand during pregnancy, and skull growth causes the scalp to expand in the fetus. Clinically, plastic and reconstructive surgeons utilize the extra skin originating from tissue expansion to reconstruct scars or repair bodily defects. Compared with traditional methods, such as skin graft and flap transfer, tissue expansion can regenerate the area of the donor tissue and substantially reduce the deformities of the donor site. In addition, expanded flaps effectively match the color and texture of the recipient area (2). Therefore, identifying hub genes and determining molecular changes in skin and soft tissue stimulated by mechanical stretch will help elucidate the biological behaviors of the cells and improve the efficiency of tissue expansion.

Recent studies have also investigated the molecular changes in expanded skin induced by mechanical stretch in animal models (3). By conducting a transcription analysis on expanded skin biopsies of pigs, Ledwon et al. (4) revealed changes in immune response activation, cell metabolism, and processes related to muscle contraction and cytoskeleton organization. Using a skin-stretched mouse model, Aragona et al. (5) showed that mechanical stretch created a transient bias in the renewal activity of epidermal stem cells and a second subpopulation of basal progenitors committed to differentiation.

However, the structure and mechanical properties of the skin in humans are different from those in other animals (6). Therefore, comprehensive molecular changes in human skin stimulated by mechanical stretch are not entirely understood. Moreover, it is difficult to collect specific human skin specimens stimulated by mechanical stretch under standard conditions and time periods. Fibroblasts are the main cell type in the dermis. Thus, human dermal fibroblasts (HDFs) are suitable cell models for studying molecular changes under mechanical stretch at distinct time periods. In this study, we focused on identifying common hub genes and their potential regulatory networks in HDFs stimulated by mechanical stretch at different stages.

## Materials and Methods

### Microarray Data

The GSE58389 gene expression dataset was downloaded from the Gene Expression Omnibus (GEO) database. The GSE58389 platform was GPL13607 (Agilent-028004 SurePrint G3 Human GE 8 × 60K Microarray). GSE58389 contained four groups (5 h control, 5 h treated, 24 h control, and 24 h treated). In each group, primary HDFs from ten donors were cultured on BIOFLEX (Ontario, Canada) culture plates and stretched for 5 or 24 h, or were left untreated as controls. Cyclic stretch was applied using the FX-4000T™ Tension Plus™ System (Flexercell International; McKeesport, PA, USA) with 16% elongation at 0.5 Hz in a half sinus regimen. Forty samples were included in the dataset (7).

### Identification of Differentially Expressed Genes

The microarray datasets at 5 and 24 h were uploaded to the interactive web tool GEO2R (https://www.ncbi.nlm.nih.gov/geo/geo2r/) to screen the DEGs in HDFs between cyclic mechanical stretching and static samples. Next, we assessed the dataset quality of the microarrays and analyzed the differences in gene expression. To maintain the number of DEGs in the original study, only genes with |log (fold change) (logFC)| >0.5 and *p* < 0.05 were selected as DEGs. To identify the common DEGs, Calculate and Draw Custom Venn Diagrams (http://bioinformatics.psb.ugent.be/webtools/Venn/) was used to obtain the overlapping DEGs in both the 5 h and 24 h groups.

### Gene Ontology and Kyoto Encyclopedia of Genes and Genomes Pathway Enrichment Analyses

Based on the web-server Metascape (https://metascape.org/gp/index.html#/main/step1), Gene Ontology (GO) and Kyoto Encyclopedia of Genes and Genomes (KEGG) pathway enrichment analyses were performed to identify the functions of DEGs in cyclic mechanical stretching and static samples at different times (8). We established the cutoff criteria as *p* < 0.01, minimum overlap genes = 3, and minimum enrichment factor >1.5. After identifying all statistically enriched GO and KEGG terms, accumulative hypergeometric *p* values and enrichment factors were calculated and used for filtering. The remaining significant terms were then hierarchically clustered into a tree based on the kappa statistical similarities among their gene memberships. Next, a kappa value of 0.3 was applied as the threshold to cast the tree into term clusters. We then selected a subset of representative terms from these clusters and converted them into a network layout.

### Construction of the Protein–Protein Interaction Network

Protein–protein interactions (PPIs) occur in all important biological processes in living organisms, such as catalyzing metabolic reactions, DNA replication, DNA transcription, responding to stimuli, and transporting molecules between locations (9). The STRING database (https://string-preview.org, version 11.5) was used to construct the PPI network of DEGs based on the default parameters (required score = 0.4; false discovery rate = 5%) (10).

### Hub Gene Analysis

Cytoscape software version 3.8.2 (Cytoscape Consortium, Boston, MA, USA) was used to visualize the PPI network, as previously described (11). The top 10 hub genes were selected using the plug-in Cytohubba within Cytoscape using the Maximal Clique Centrality method and were then displayed in the extended subnetwork (12). Further GO analyses of hub genes were performed using Metascape.

### Regulatory Network of Hub Genes

The NetworkAnalyst web tool (https://www.networkanalyst.ca) was used to predict miRNA gene and gene transcription factor (TF) interactions (13). Visualization of two regulatory hub gene networks was also conducted using Cytoscape.

### Statistical Analysis

Descriptive data are presented as the mean ± SD, median, or frequencies and proportions, as appropriate. Statistical significance was set at *p* < 0.05. All statistical analyses were performed using SPSS version 25.0 (IBM Corporation, Armonk, NY, USA).

## Results

### Screening of DEGs

**Figure 1:**
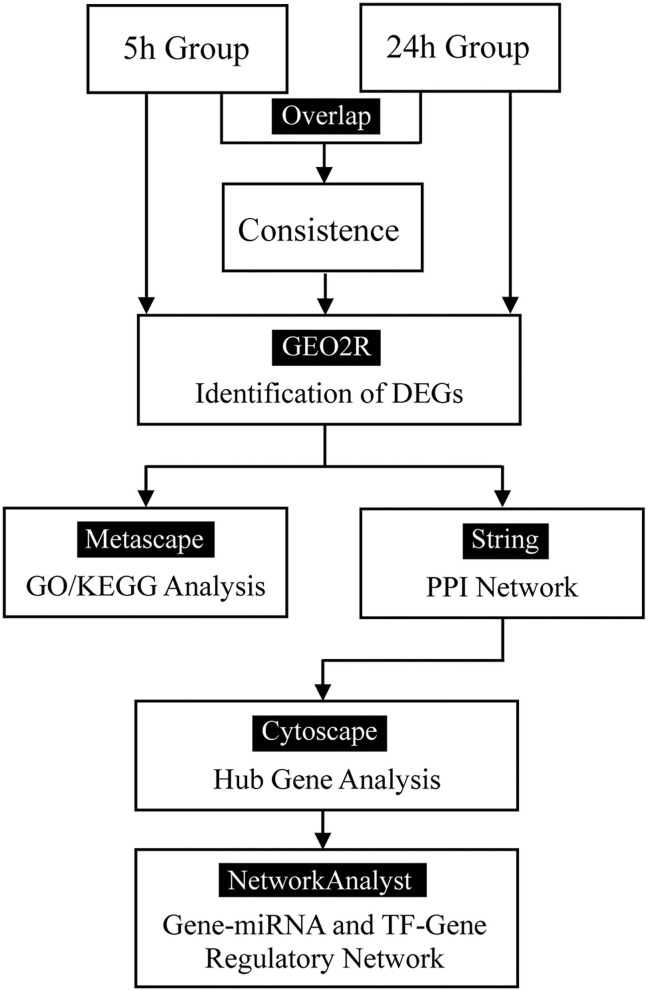
Flow diagram.

**Figure 2:**
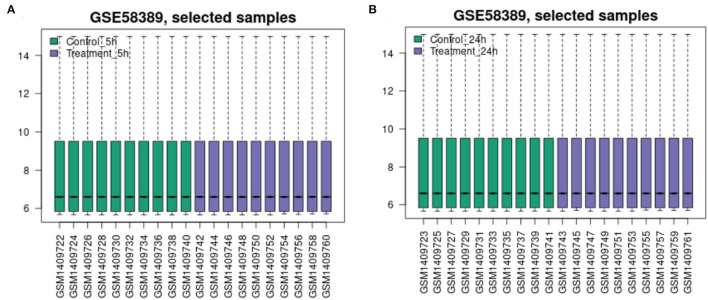
Data quality boxplot. **(A)** At 5 h and **(B)** at 24 h.

**Figure 3:**
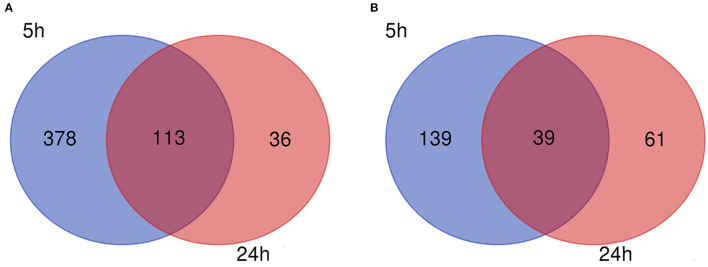
Venn diagram. **(A)** Downregulated and **(B)** upregulated genes.

The flow diagram created in this study is shown in Figure 1. The boxplots in Figure 2 show the homogeneity of data quality after GEO2R analyses. According to the selection criteria, 669 DEGs were identified at the early stage (5 h), namely, 491 downregulated and 178 upregulated genes, and 249 DEGs were identified at the late stage (24 h), namely, 149 downregulated and 100 upregulated genes. As shown in Figure 3, the Venn diagrams demonstrate overlap in DEGs in both the early and late stages, indicating that there are common DEGs under continuous mechanical stretch.

### GO and KEGG Pathway Enrichment Analyses

**Figure 4:**
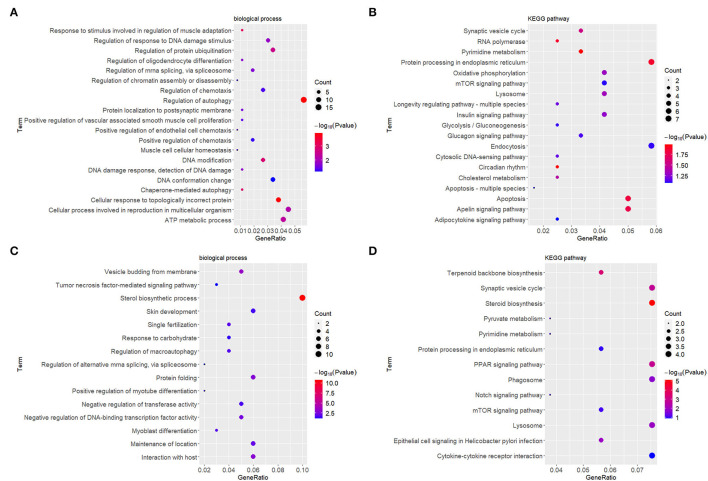
GO enrichment and KEGG analyses showed changes in the molecular response and signaling pathway of HDFs under mechanical stretch. **(A)** GO enrichment analysis of the DEGs at 5 h and **(B)** at 24 h. **(C)** KEGG analysis of the DEGs at 5 h and **(D)** at 24 h. The gene ratio indicates the number of DEGs associated with the GO term divided by the total number of DEGs. The size of the dots represents the number of DEGs associated with the GO term, and the color represents the negative value of log_10_ of *p* value. GO, Gene Ontology; KEGG, Kyoto Encyclopedia of Genes and Genomes; HDFs, human dermal fibroblasts; DEGs, differentially expressed genes.

At the early stage (5 h), the top enriched GO term was “regulation of autophagy” (GO:0010506), and the top KEGG term was “pyrimidine metabolism” (hsa00240). At the late stage (24 h), the top GO term was “sterol biosynthetic process” (GO:0016126), and the top KEGG term was “synaptic vesicle cycle” (ko04721). Genes within each cluster in networks at different stages are shown in Supplementary Table 1. The enrichments and number of DEGs in each statistically significant term are depicted by bubble plots in Figure 4, and the corresponding data are shown in Supplementary Table 2.

### PPI Networks and Hub Genes

**Figure 5:**
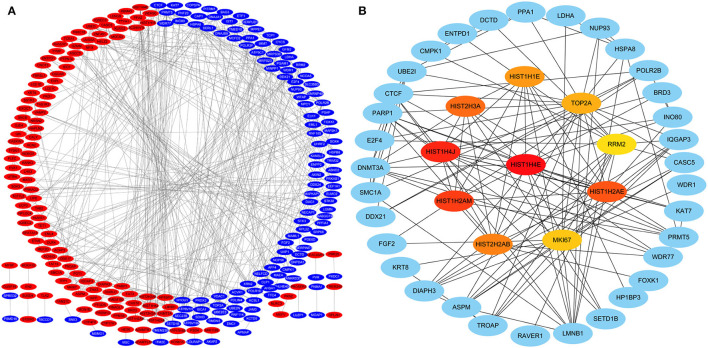
PPI network at 5 h. **(A)** Entire network. Downregulated and upregulated genes are represented by red and blue backgrounds, respectively. **(B)** Subnetwork for the top 10 hub genes and neighbors. The backgrounds of hub genes are colored from red to yellow by rank, and the backgrounds of neighboring genes are light blue. PPI, protein-protein interactions.

**Figure 6:**
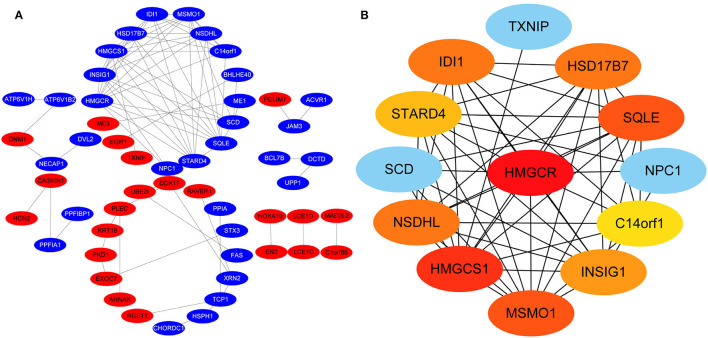
PPI network at 24 h. **(A)** Entire network. Downregulated and upregulated genes are represented by red and blue backgrounds, respectively. **(B)** Subnetwork for the top 10 hub genes and neighbors. The backgrounds of hub genes are colored from red to yellow by rank, and the backgrounds of neighboring genes are light blue. PPI, protein-protein interactions.

**Figure 7:**
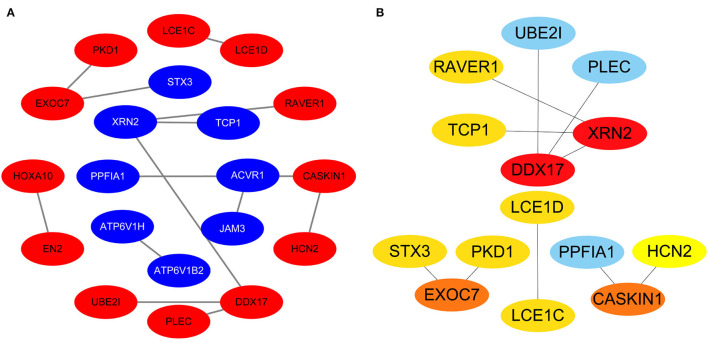
Common PPI network. **(A)** Entire network. Downregulated and upregulated genes are represented by red and blue backgrounds, respectively. **(B)** Subnetwork for the top 10 hub genes and neighbors. The backgrounds of hub genes are colored from red to yellow by rank, and the backgrounds of neighboring genes are light blue. PPI, protein-protein interactions.

Protein-protein interactions (PPIs) were obtained using STRING, visualized using Cytoscape, and further analyzed using the plug-in Cytohubba. Early (5 h), late (24 h), and common PPI networks are shown in Figures 5A, 6A, 7A, respectively. The hub genes at different stages are listed in Table 1. Subnetworks for the top 10 hub genes and their neighbors are illustrated in Figures 5B, 6B, 7B. GO hub gene analyses indicated entirely different terms at different stages (Table 2). Notably, hub genes related to skin development (*PKD1, LCE1C*, and *LCE1D*) commonly changed under mechanical stretch.

**Table 1:** Early (5 h), later (24 h) and consistent top 10 hub Genes.

**Stage**	**Top 10 hub genes^a^**	
	**Downregulated**	**Upregulated**
Early	*HIST1H4E (H4C5), HIST1H4J (H4C11), HIST1H2AM (H2AC17), HIST1H2AE (H2AC8), HIST2H3A (H3C15), HIST2H2AB (H2AC21), HIST1H1E (H1-4), MKI67*	*TOP2A, RRM2*
Later	*None*	*HMGCR, HMGCS1, SQLE, MSMO1, HSD17B7, IDI1, NSDHL, INSIG1, STARD4, C14orf1 (ERG28)*
Consistent	*DDX17, EXOC7, CASKIN1, RAVER1, LCE1D, LCE1C, PKD1*	*XRN2, TCP1, STX3*

*^a^The genes were ranked by Maximal Clique Centrality method*.

**Table 2:** GO^*^ terms of hub genes.

**Stage**	**Term description**	**Gene**	***p* value**
Early	DNA packaging	*HIST1H1E, TOP2A, HIST1H4J, HIST1H4E, HIST2H3A*	8.30E−09
	Chromatin silencing	*HIST1H1E, HIST1H2AE, HIST1H2AM, HIST2H2AB*	1.02E−08
Late	Sterol biosynthetic process	*HMGCR, HMGCS1, IDI1, INSIG1, MSMO1, SQLE, C14orf1, NSDHL, HSD17B7, STARD4*	1.04E−23
	Steroid biosynthetic process	*HMGCR, HMGCS1, IDI1, INSIG1, MSMO1, SQLE, C14orf1, NSDHL, HSD17B7, STARD4*	6.24E−23
	Isoprenoid biosynthetic process	*HMGCR, HMGCS1, IDI1, INSIG1*	1.05E−07
	Cellular ketone metabolic process	*HMGCR, INSIG1, STARD4, SQLE, MSMO1*	4.59E−05
Common	**Skin development**	*PKD1, LCE1C, LCE1D*	9.21E−05
	mRNA processing	*DDX17, XRN2, RAVER1*	6.08E−04

### Gene-miRNA and TF-Gene Regulatory Network

**Figure 8:**
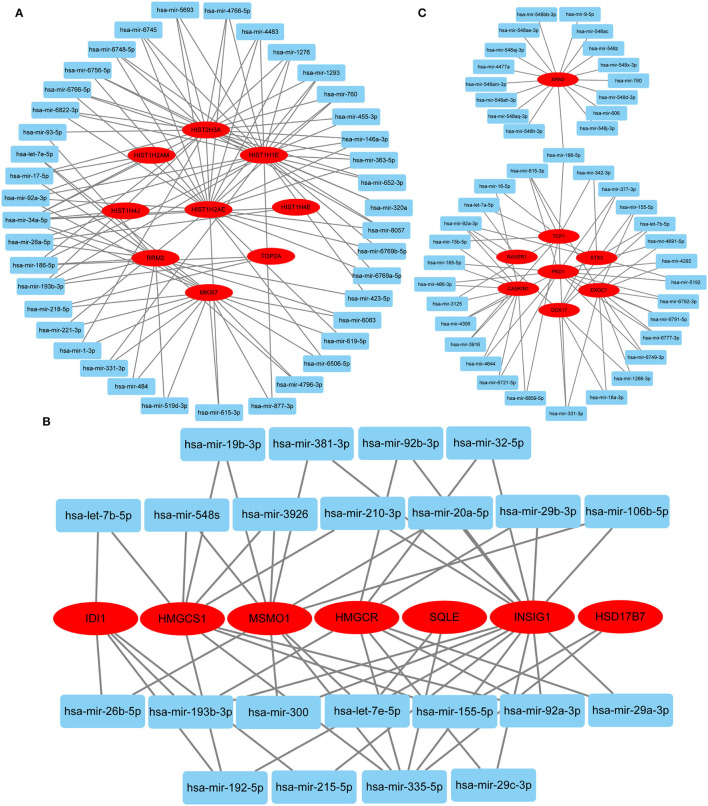
Gene-miRNA regulatory network. **(A)** Early stage (5 h); **(B)** late stage (24 h); **(C)** common stage. The background colors of genes and miRNAs are red and light blue, respectively. The sole connection of the gene-miRNA is hidden.

**Figure 9:**
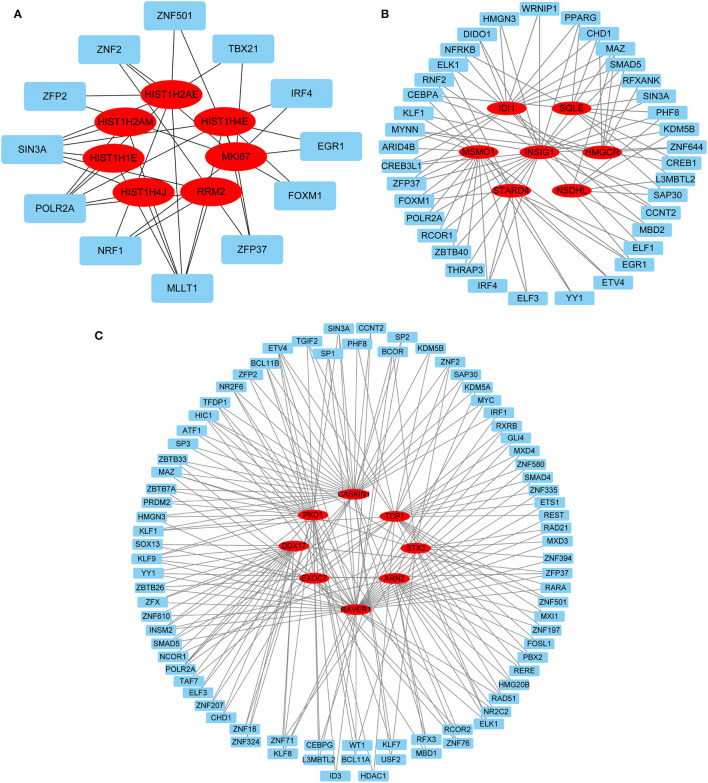
TF-gene regulatory network. **(A)** Early stage (5 h); **(B)** late stage (24 h); **(C)** common stage. The background colors of genes and miRNAs are red and light blue, respectively. The sole connection of the TF-gene is hidden. TF, transcriptional factor.

Hub gene-related miRNAs or TFs were ranked using the Maximal Clique Centrality method. The top three miRNAs or TFs closest to hub genes are listed in Table 3. Notably, certain miRNAs and TFs were predictably related to hub genes in at least two different stages. With regard to miRNAs, hsa-mir-92a-3p was present at all stages, and hsa-mir-193b-3p appeared at the early and late stages. For TFs, POLR2A belonged to early and common hub genes, SMAD5 was part of the early and late hub genes, and MAZ pertained to late and common hub genes. The gene-miRNA regulatory networks are shown in Figure 8. The TF-gene regulatory networks are illustrated in Figure 9.

**Table 3:** Top 3 miRNAs or TFs closest to hub genes^a^.

**Stage**	**miRNAs**	**TFs**
Early	hsa-mir-34a-5p, **hsa-mir-92a-3p**, **hsa-mir-193b-3p**^b^	*SIN3A, **POLR2A***, **SMAD5**^c^
Late	hsa-mir-335-5p, **hsa-mir-193b-3p**, **hsa-mir-92a-3p**^d^	***MAZ**, CEBPA*, **SMAD5**^e^
Common	**hsa-mir-92a-3p**, hsa-mir-615-3p, hsa-mir-331-3p	*ETV4, KLF9*, **POLR2A**^f^

*^a^The miRNAs or TFs were ranked using the Maximal Clique Centrality method*.

b *Score of hsa-mir-186-5p and hsa-mir-17-5p was equal to the last one (score = 4)*.

c *Score of MLLT1 was equal to the last one (score = 7)*.

d *Score of hsa-mir-192-5p were equal to the last one (score = 3)*.

e *Score of CHD1, MYNN, PPARG, CREB3L1, ZFP37, FOXM1, KDM5B, PHF8, IRF4, SAP30 and EGR1 was equal to the last one (score = 3)*.

f *Score of MAZ, HIC1, INSM2, KLF8 and ELK1 was equal to the last one (score = 4)*.

*Bold value means that one miRNA or TF appears in at least two stages*.

## Discussion

Mechanical stretch-induced skin regeneration provides sufficient material for wound repair and organ reconstruction. However, the mechanisms by which mechanical stretch promotes skin regeneration remain predominantly unknown. Fibroblasts are one of the main cell types present in the skin; thus, determining the effects of mechanical stretch on HDFs may help to improve our understanding of the mechanisms and assist in promoting expanded skin regeneration. Reichenbach et al. (7) compared stretched HDFs with unstretched HDFs to identify DEGs using mRNA microarray data. However, because of unknown reasons, these authors did not further study or discuss the functions of these DEGs. More crucially, the GO and KEGG pathway enrichment analyses, PPI network construction, hub gene analysis, and regulatory hub gene network were absent in the previous work (7). To solve this problem, we performed this study based on the uploaded mRNA microarray data and previous reports (7). Herein, we provide the DEGs in mechanically stretched HDFs and unstretched HDFs, and the common DEGs present in both the early and late stages. More importantly, we presented novel results of GO and KEGG pathway enrichment analyses, PPI network construction, hub gene analysis, and a regulatory hub gene network. These may assist in identifying genes involved in promoting fibroblast proliferation and skin regeneration.

### GO and KEGG Analyses

Gene Ontology (GO) and KEGG analyses can help us elucidate important biological functions and pathways involving DEGs. At the early stage, the top enriched GO term was determined to be “regulation of autophagy.” Consistently, in our previous study, autophagosomes containing mitochondria and cytoplasm were clearly observed in expanded murine scalp fibroblasts at the early stage (14). This suggests that autophagy of HDFs may occur early in response to mechanical stretch. The top KEGG term was “pyrimidine metabolism,” demonstrating that HDFs initiated active nucleotide synthesis. At the late stage, the top GO term was “sterol biosynthetic process,” representing changes in energy metabolism. The top KEGG term was “synaptic vesicle cycle,” representing substance and signal exchanges through repeated exocytosis and endocytosis. However, the GO and KEGG analyses above only provided a general explanation of molecular changes and did not consider the complex interactions between molecules. Therefore, we constructed PPI networks and identified hub genes at different stages.

### Early Hub Genes

Following a 5-h cyclic mechanical stretch, representing the early stage, the top 10 hub genes identified primarily participated in DNA and chromatin alterations. Seven histone-related genes were identified and downregulated at this stage: H4 clustered histone 5 (*HIST1H4E, H4C5*), H4 clustered histone 11 (*HIST1H4J, H4C11*), H2A clustered histone 17 (*HIST1H2AM, H2AC17*), H2A clustered histone 8 (*HIST1H2AE, H2AC8*), H3 clustered histone 15 (*HIST2H3A, H3C15*), H2A clustered histone 21 (*HIST2H2AB, H2AC21*), and cluster member H1.4 linker histone (*HIST1H1E, H1-4*). Histones are fundamental structural components of chromatin (15). Eukaryotic DNA is wound around an octamer of core histones H2A, H2B, H3, and H4. The binding of the linker histone H1 promotes higher-order chromatin organization (15). The marker of proliferation Ki-67 gene (*MKI67*) encodes a nuclear protein that is required to maintain individual mitotic chromosomes dispersed in the cytoplasm following nuclear envelope disassembly and may be necessary for cell proliferation (16). DNA topoisomerase II alpha (*TOP2A*) encodes a key decatenating enzyme that alters DNA topology by binding to two double-stranded DNA molecules (17). *TOP2A* is generally upregulated in proliferating cells (18). However, in another study, fibroblasts, such as mouse NIH 3T3 and 3T6 cells, did not show high *TOP2A* expression (19). Even under certain stimuli (radiation or drugs), *TOP2A* expression is downregulated when fibroblasts maintain proliferation activity (20, 21). This may be related to the negative feedback regulation in fibroblasts, which prevents excessive cell proliferation (22). Another upregulated gene, ribonucleotide reductase regulatory subunit M2 (*RRM2*), encodes one of two non-identical subunits for ribonucleotide reductase, which is necessary for DNA synthesis. *RRM2* also functions as a downstream factor of β-catenin as an inhibitor of Wnt signaling (23), and β-catenin activation can stimulate fibroblast proliferation (24, 25).

### Late Hub Genes

Following a 24-h cyclic mechanical stretch, representing the late stage, the top 10 hub genes identified primarily participated in cholesterol metabolism. 3-hydroxy-3-methylglutaryl-CoA synthase 1 catalyzes the condensation of acetyl-CoA with acetoacetyl-CoA to form (3S)-hydroxy-3-methylglutaryl-CoA (*HMG-CoA*), which is then converted by HMG-CoA reductase into mevalonate, a precursor for cholesterol synthesis (26). Next, mevalonate is converted into lanosterol under the action of various enzymes, namely, isopentenyl-diphosphate delta isomerase 1 and squalene epoxidase (26). Lanosterol can then be diverted into either the Bloch pathway, producing cholesterol via desmosterol, or the Kandutsch–Russell pathway, *via* 7-dehydrocholesterol. Methylsterol monooxygenase 1, 17-betahydroxysteroid dehydrogenase 7, and NAD(*P*)-dependent steroid dehydrogenase-like protein are involved in these two pathways (26). Furthermore, *INSIG1, STARD4*, and *C14orf1* assist in controlling sterol biosynthesis (27–29). Cholesterol is a critical regulator of lipid bilayer dynamics and plasma membrane organization in eukaryotes (30). The physical properties of the membranes depend on lipid composition; the stiffness and fluidity of the bilayers are essentially determined by the sterol content (31). Various ion channels are modulated by cellular cholesterol and partitioned into cholesterol-enriched membrane rafts (32). After cholesterol depletion, inhibition of stretch-activated cation channels is mediated *via* actin remodeling and is initiated by the disruption of lipid rafts (31). Thus, cell membrane cholesterol reduction is closely related to changes in cell morphology after stretching. Alterations in metabolic processes play a role in regulating inflammation and extracellular matrix deposition (33). In summary, changes in cholesterol metabolism are an important biological feature of HDFs under stretch conditions at the late stage and may become a potential target to help HDFs adapt more rapidly to changing environments.

### Common Hub Genes

Using overlapping DEGs between 5 and 24 h, the consistent top 10 hub genes (seven downregulated and three upregulated) were determined to be different at both stages. This shows that the biological effects of mechanical stretch varied over time. *DDX17, XRN2*, and *STX3* are involved in transcriptional regulation. *DDX17* encodes an important context-dependent transcriptional regulator that promotes cell growth by interacting with estrogen receptors (34). *XRN2* (*DHP1* in the yeast genus Schizosaccharomyces), an upregulated gene, triggers premature transcription termination and nucleates heterochromatin to promote meiotic gene silencing (35). *STX3*, another upregulated gene, also acts as a transcriptional regulator; inhibition of endogenous *STX3* expression alters cellular genes and promotes cell proliferation (36). *EXOC7, CASKIN1, RAVER1*, and *TCP1* are involved in cytoskeleton rearrangement. *EXOC7*, in addition to functioning in exocytosis, regulates actin at the leading edges of migrating cells, thereby coordinating cytoskeleton and membrane trafficking during cell migration (37). *CASKIN1*, a scaffold protein, regulates actin filaments (38). *RAVER1* interacts with the cytoskeletal proteins actinin and vinculin (39). *TCP1*, another upregulated gene, inhibits the transformation of fibroblasts into myofibroblasts, thus adjusting for the morphological changes caused by mechanical stretch (40). Previous studies have also reported that changes in the cytoskeleton are important results of mechanical signals and mediate the synthesis of the extracellular matrix triggered by mechanical stretch (41). Overall, hub genes related to transcriptional regulation and cytoskeleton rearrangement are potential targets for promoting HDF regeneration and alignment.

Importantly, three consistent DEGs (*LCE1D, LCE1C*, and *PKD1*) were downregulated and identified as hub genes. These are all closely related to skin development, according to the GO analysis. Mechanical stretch is believed to regulate intracellular calcium homeostasis, resulting in changes in a series of downstream signaling pathways (42). *LCE1D* and *LCE1C* may be downregulated in response to extracellular calcium alterations (43). However, previous studies on the function of late cornified envelope proteins were mainly conducted in keratinocytes and not in fibroblasts. Thus, the mechanisms by which *LCE1D* and *LCE1C* are involved in fibroblast differentiation and growth may be new targets for future research. *PKD1* encodes an integral membrane protein involved in the regulation of mechanotransduction signaling (44). The component of heteromeric calcium-permeable ion channels formed by *PKD1* and *PKD2* is activated by the interaction between *PKD1* and a Wnt family member, such as *WNT3A* or *WNT9B* (45). *PKD1* induces cell migration by regulating rearrangements and cell-cell mechanical adhesion (46), inhibits cell apoptosis through a PKR-eIF2α pathway (47), and regulates the cell cycle by inhibiting DNA binding (48). Therefore, downregulation of *PKD1* may be an important mechanism in HDF-sensing mechanical stretching and controlling cell growth and differentiation.

### Regulatory Network

Predicting gene-miRNA and TF-gene regulatory networks at different stages can provide a reference for subsequent interventions. Two miRNAs (hsa-mir-92a-3p and hsa-mir-193b-3p) and three TFs (POLR2A, SMAD5, and MAZ) were identified in at least two stages. Hsa-mir-92a-3p is extensively involved in the regulation of cellular proliferation and angiogenesis but has cell type-specific effects *in vivo* (49, 50). Hsa-mir-193b-3p is widely expressed in normal human tissues and displays antiproliferative effects associated with its functions in different cell differentiation processes (51). POLR2A encodes the largest subunit of RNA polymerase II, which is responsible for synthesizing messenger RNA in eukaryotes. Multiple pathways have been shown to regulate cell proliferation by affecting POLR2A expression (52–54). SMAD5 proteins are intracellular signaling molecules that mediate canonical bone morphogenetic protein pathways and are involved in tissue regeneration (55). MAZ is a zinc finger transcription factor that activates the expression of tissue-specific genes and represses the expression of the c-myc proto-oncogene. Previous data indicate that MAZ is a growth suppressor protein that affects the cell cycle in fibroblasts (56). Therefore, *MAZ* knockdown may be effective in continuously activating HDFs. The above regulatory network provides a reference for further intervention approaches.

## Conclusion

At the early stage, DNA and chromatin alterations were clearly observed; at the late stage, cholesterol metabolism was strengthened to adapt to the changing environment. In common stages, genes related to transcriptional regulation, cytoskeleton rearrangement, and skin development were found to be potential targets to promote HDF growth and alignment under mechanical stretch. The hub genes and their regulatory networks were different during different periods.

We found that hub genes and their functions were considerably different during distinct periods. Bioinformatics analysis of common hub genes in stretched HDFs present at different stages provides direction for subsequent research. Thus, our findings can provide a reference for the precise regulation of HDF behavior in response to mechanical stretch. In the future, *PKD1*, among other common hub genes, maybe a new target to promote mechanical stretch-induced skin regeneration.

## Data Availability Statement

The datasets presented in this study can be found in online repositories. The names of the repository/repositories and accession number(s) can be found in the article/Supplementary Material.

## Author Contributions

CD and WL: designing the study, conducting the study, acquiring and analyzing data, and writing the manuscript. YZ, YS, JD, ZH, and TW: the concept of the study, analyzing data, and editing the manuscript. YZ and XM: concept of the study, designing the study, and writing/editing the manuscript. All authors contributed to the article and approved the submitted version.

## Funding

Funding from the National Natural Science Foundation of China (82172229 and 81971851), the Natural Science Basic Research Plan in Shaanxi Province of China (2022JM-600), and the Foundation of Xijing Hospital Grant (XJZT21CM33) was received for this study.

## Conflict of Interest

The authors declare that the research was conducted in the absence of any commercial or financial relationships that could be construed as a potential conflict of interest.

## Publisher's Note

All claims expressed in this article are solely those of the authors and do not necessarily represent those of their affiliated organizations, or those of the publisher, the editors and the reviewers. Any product that may be evaluated in this article, or claim that may be made by its manufacturer, is not guaranteed or endorsed by the publisher.

## References

[1] Silver FH Siperko LM Seehra GP. Mechanobiology of force transduction in dermal tissue. Skin Res Technol. (2003) 9:3–23. 10.1034/j.1600-0846.2003.00358.x 12535279

[2] Dong C Zhu M Huang L Liu W Liu H Jiang K. Risk factors for tissue expander infection in scar reconstruction: a retrospective cohort study of 2374 consecutive cases. Burns Trauma. (2021) 8:tkaa037. 10.1093/burnst/tkaa037 33426134 PMC7780061

[3] Topczewska JM Ledwon JK Vaca EE Gosain AK. Mechanical stretching stimulates growth of the basal layer and rete ridges in the epidermis. J Tissue Eng Regen Med. (2019) 13:2121–5. 10.1002/term.2952 31381259 PMC6872913

[4] Ledwon JK Kelsey LJ Vaca EE Gosain AK. Transcriptomic analysis reveals dynamic molecular changes in skin induced by mechanical forces secondary to tissue expansion. Sci Rep. (2020) 10:15991. 10.1038/s41598-020-71823-z 32994433 PMC7524724

[5] Aragona M Sifrim A Malfait M Song Y Van Herck J Dekoninck S. Mechanisms of stretch-mediated skin expansion at single-cell resolution. Nature. (2020) 584:268–73. 10.1038/s41586-020-2555-7 32728211 PMC7116042

[6] Zomer HD Trentin AG. Skin wound healing in humans and mice: challenges in translational research. J Dermatol Sci. (2018) 90:3–12. 10.1016/j.jdermsci.2017.12.009 29289417

[7] Reichenbach M Reimann K Reuter H. Gene expression in response to cyclic mechanical stretch in primary human dermal fibroblasts. Genom Data. (2014) 2:335–9. 10.1016/j.gdata.2014.09.010 26484124 PMC4535970

[8] Zhou Y Zhou B Pache L Chang M Khodabakhshi AH Tanaseichuk O. Metascape provides a biologist-oriented resource for the analysis of systems-level datasets. Nat Commun. (2019) 10:1523. 10.1038/s41467-019-09234-6 30944313 PMC6447622

[9] Peng X Wang J Peng W Wu FX Pan Y. Protein-protein interactions: detection, reliability assessment and applications. Brief Bioinform. (2017) 18:798–819. 10.1093/bib/bbw066 27444371

[10] Szklarczyk D Gable AL Nastou KC Lyon D Kirsch R Pyysalo S. The STRING database in 2021: customizable protein-protein networks, and functional characterization of user-uploaded gene/measurement sets. Nucleic Acids Res. (2021) 49:D605–605D612. 10.1093/nar/gkaa1074 33237311 PMC7779004

[11] Shannon P Markiel A Ozier O Baliga NS Wang JT Ramage D. Cytoscape: a software environment for integrated models of biomolecular interaction networks. Genome Res. (2003) 13:2498–504. 10.1101/gr.1239303 14597658 PMC403769

[12] Chin CH Chen SH Wu HH Ho CW Ko MT Lin CY. cytoHubba: identifying hub objects and sub-networks from complex interactome. BMC Syst Biol. (2014) 8 Suppl 4:S11. 10.1186/1752-0509-8-S4-S11 25521941 PMC4290687

[13] Zhou G Soufan O Ewald J Hancock R Basu N Xia J. NetworkAnalyst 3.0: a visual analytics platform for comprehensive gene expression profiling and meta-analysis. Nucleic Acids Res. (2019) 47:W234–41. 10.1093/nar/gkz240 30931480 PMC6602507

[14] Yu Z Liu S Cui J Song Y Wang T Song B. Early histological and ultrastructural changes in expanded murine scalp. Ultrastruct Pathol. (2020) 44:141–52. 10.1080/01913123.2020.1720876 31989853

[15] Biterge B Schneider R. Histone variants: key players of chromatin. Cell Tissue Res. (2014) 356:457–66. 10.1007/s00441-014-1862-4 24781148

[16] Cuylen S Blaukopf C Politi AZ Müller-Reichert T Neumann B Poser I. Ki-67 acts as a biological surfactant to disperse mitotic chromosomes. Nature. (2016) 535:308–12. 10.1038/nature18610 27362226 PMC4947524

[17] Lee S Jung SR Heo K Byl JA Deweese JE Osheroff N. DNA cleavage and opening reactions of human topoisomerase IIα are regulated via Mg2+-mediated dynamic bending of gate-DNA. Proc Natl Acad Sci U S A. (2012) 109:2925–30. 10.1073/pnas.1115704109 22323612 PMC3286967

[18] Liu T Zhang H Yi S Gu L Zhou M. Mutual regulation of MDM4 and TOP2A in cancer cell proliferation. Mol Oncol. (2019) 13:1047–58. 10.1002/1878-0261.12457 30672125 PMC6487731

[19] Hsiang YH Wu HY Liu LF. Proliferation-dependent regulation of DNA topoisomerase II in cultured human cells. Cancer Res. (1988) 48:3230–5. 2835157

[20] de Toledo SM Azzam EI Keng P Laffrenier S Little JB. Regulation by ionizing radiation of CDC2, cyclin A, cyclin B, thymidine kinase, topoisomerase IIalpha, and RAD51 expression in normal human diploid fibroblasts is dependent on p53/p21Waf1. Cell Growth Differ. (1998) 9:887–96. 9831241

[21] Janikowska G Kurzeja E Janikowski M Strzałka-Mrozik B Pyka-Pajak A. Janikowski T. The effect of cyclosporine A on dermal fibroblast cell - transcriptomic analysis of inflammatory response pathway. Curr Pharm Biotechnol. (2020) 21:1213–23. 10.2174/1389201021666200416103928 32297577

[22] Gao XH Li J Liu Y Liu QZ Hao LQ Liu LJ. ZNF148 modulates TOP2A expression and cell proliferation via ceRNA regulatory mechanism in colorectal cancer. Medicine. (2017) 96:e5845. 10.1097/MD.0000000000005845 28072746 PMC5228706

[23] Tang LY Deng N Wang LS Dai J Wang ZL Jiang XS. Quantitative phosphoproteome profiling of Wnt3a-mediated signaling network: indicating the involvement of ribonucleoside-diphosphate reductase M2 subunit phosphorylation at residue serine 20 in canonical Wnt signal transduction. Mol Cell Proteomics. (2007) 6:1952–67. 10.1074/mcp.M700120-MCP200 17693683

[24] Collins CA Kretzschmar K Watt FM. Reprogramming adult dermis to a neonatal state through epidermal activation of β-catenin. Development. (2011) 138:5189–99. 10.1242/dev.064592 22031549 PMC3210498

[25] Driskell RR Lichtenberger BM Hoste E Kretzschmar K Simons BD Charalambous M. Distinct fibroblast lineages determine dermal architecture in skin development and repair. Nature. (2013) 504:277–81. 10.1038/nature12783 24336287 PMC3868929

[26] Sharpe LJ Brown AJ. Controlling cholesterol synthesis beyond 3-hydroxy-3-methylglutaryl-CoA reductase (HMGCR). J Biol Chem. (2013) 288:18707–15. 10.1074/jbc.R113.479808 23696639 PMC3696645

[27] Rodriguez-Agudo D Calderon-Dominguez M Ren S Marques D Redford K Medina-Torres MA. Subcellular localization and regulation of StarD4 protein in macrophages and fibroblasts. Biochim Biophys Acta. (2011) 1811:597–606. 10.1016/j.bbalip.2011.06.028 21767660 PMC3156897

[28] Johnson BM DeBose-Boyd RA. Underlying mechanisms for sterol-induced ubiquitination and ER-associated degradation of HMG CoA reductase. Semin Cell Dev Biol. (2018) 81:121–8. 10.1016/j.semcdb.2017.10.019 29107682 PMC6341991

[29] Ke X Xia XY Zheng RC Zheng YG. Identification of a consensus motif in Erg28p required for C-4 demethylation in yeast ergosterol biosynthesis based on mutation analysis, *FEMS Microbiol Lett*. (2018) 365:fny002. 10.1093/femsle/fny002 29319811

[30] Brown DA London E. Structure and function of sphingolipid- and cholesterol-rich membrane rafts. J Biol Chem. (2000) 275:17221–4. 10.1074/jbc.R000005200 10770957

[31] Chubinskiy-Nadezhdin VI Negulyaev YA Morachevskaya EA. Cholesterol depletion-induced inhibition of stretch-activated channels is mediated via actin rearrangement. Biochem Biophys Res Commun. (2011) 412:80–5. 10.1016/j.bbrc.2011.07.046 21798240

[32] Levitan I Fang Y Rosenhouse-Dantsker A Romanenko V. Cholesterol and ion channels. Subcell Biochem. (2010) 51:509–49. 10.1007/978-90-481-8622-8_19 20213557 PMC2895485

[33] Zhao X Psarianos P Ghoraie LS Yip K Goldstein D Gilbert R. Metabolic regulation of dermal fibroblasts contributes to skin extracellular matrix homeostasis and fibrosis. Nat Metab. (2019) 1:147–57. 10.1038/s42255-018-0008-5 32694814

[34] Wortham NC Ahamed E Nicol SM Thomas RS Periyasamy M Jiang J. The DEAD-box protein p72 regulates ERalpha-/oestrogen-dependent transcription and cell growth, and is associated with improved survival in ERalpha-positive breast cancer. Oncogene. (2009) 28:4053–64. 10.1038/onc.2009.261 19718048 PMC2780396

[35] Chalamcharla VR Folco HD Dhakshnamoorthy J Grewal SI. Conserved factor Dhp1/Rat1/Xrn2 triggers premature transcription termination and nucleates heterochromatin to promote gene silencing. Proc Natl Acad Sci U S A. (2015) 112:15548–55. 10.1073/pnas.1522127112 26631744 PMC4697380

[36] Giovannone AJ Winterstein C Bhattaram P Reales E Low SH Baggs JE. Soluble syntaxin 3 functions as a transcriptional regulator. J Biol Chem. (2018) 293:5478–91. 10.1074/jbc.RA117.000874 29475951 PMC5900775

[37] Zuo X Zhang J Zhang Y Hsu SC Zhou D Guo W. Exo70 interacts with the Arp2/3 complex and regulates cell migration. Nat Cell Biol. (2006) 8:1383–8. 10.1038/ncb1505 17086175

[38] Koprivanacz K Toke O Besztercei B Juhász T Radnai L Mero B. The SH3 domain of Caskin1 binds to lysophosphatidic acid suggesting a direct role for the lipid in intracellular signaling. Cell Signal. (2017) 32:66–75. 10.1016/j.cellsig.2017.01.019 28104445

[39] Gromak N Rideau A Southby J Scadden AD Gooding C Hüttelmaier S. The PTB interacting protein raver1 regulates alpha-tropomyosin alternative splicing. EMBO J. (2003) 22:6356–64. 10.1093/emboj/cdg609 14633994 PMC291850

[40] Darden DL Hu FZ Ehrlich MD Gorry MC Dressman D Li HS. RNA differential display of scarless wound healing in fetal rabbit indicates downregulation of a CCT chaperonin subunit and upregulation of a glycophorin-like gene transcript. J Pediatr Surg. (2000) 35:406–19. 10.1016/s0022-3468(00)90204-5 10726679

[41] Chiquet M Tunç-Civelek V Sarasa-Renedo A. Gene regulation by mechanotransduction in fibroblasts. Appl Physiol Nutr Metab. (2007) 32:967–73. 10.1139/H07-053 18059623

[42] Razzak MA Hossain MS Radzi ZB Yahya NA Czernuszka J Rahman MT. Cellular and molecular responses to mechanical expansion of tissue. Front Physiol. (2016) 7:540. 10.3389/fphys.2016.00540 27899897 PMC5111402

[43] Jackson B Tilli CM Hardman MJ Avilion AA MacLeod MC Ashcroft GS. Late cornified envelope family in differentiating epithelia–response to calcium and ultraviolet irradiation. J Invest Dermatol. (2005) 124:1062–70. 10.1111/j.0022-202X.2005.23699.x 15854049

[44] Besschetnova TY Kolpakova-Hart E Guan Y Zhou J Olsen BR Shah JV. Identification of signaling pathways regulating primary cilium length and flow-mediated adaptation. Curr Biol. (2010) 20:182–7. 10.1016/j.cub.2009.11.072 20096584 PMC2990526

[45] Kim S Nie H Nesin V Tran U Outeda P Bai CX. The polycystin complex mediates Wnt/Ca(2+) signaling. Nat Cell Biol. (2016) 18:752–64. 10.1038/ncb3363 27214281 PMC4925210

[46] Boca M D'Amato L Amato L Distefano G Polishchuk RS Germino GG. Polycystin-1 induces cell migration by regulating phosphatidylinositol 3-kinase-dependent cytoskeletal rearrangements and GSK3beta-dependent cell cell mechanical adhesion. Mol Biol Cell. (2007) 18:4050–61. 10.1091/mbc.e07-02-0142 17671167 PMC1995705

[47] Tang Y Wang Z Yang J Zheng W Chen D Wu G. Polycystin-1 inhibits eIF2α phosphorylation and cell apoptosis through a PKR-eIF2α pathway. Sci Rep. (2017) 7:11493. 10.1038/s41598-017-11526-0 28904368 PMC5597606

[48] Li X Luo Y Starremans PG McNamara CA Pei Y Zhou J. Polycystin-1 and polycystin-2 regulate the cell cycle through the helix-loop-helix inhibitor Id2. Nat Cell Biol. (2005) 7:1202–12. 10.1038/ncb1326 16311606

[49] Rogg EM Abplanalp WT Bischof C John D Schulz MH Krishnan J. Analysis of cell type-specific effects of microRNA-92a provides novel insights into target regulation and mechanism of action. Circulation. (2018) 138:2545–58. 10.1161/CIRCULATIONAHA.118.034598 30571345

[50] Li JL Luo P. MiR-140-5p and miR-92a-3p suppress the cell proliferation, migration and invasion and promoted apoptosis in Wilms' tumor by targeting FRS2. Eur Rev Med Pharmacol Sci. (2020) 24:97–108. 10.26355/eurrev_202001_19899 31957822

[51] Khordadmehr M Shahbazi R Sadreddini S Baradaran B. miR-193: A new weapon against cancer. J Cell Physiol. (2019) 234:16861–72. 10.1002/jcp.28368 30779342

[52] Yamada K Hayashi M Madokoro H Nishida H Du W Ohnuma K. Nuclear localization of CD26 induced by a humanized monoclonal antibody inhibits tumor cell growth by modulating of POLR2A transcription. PLoS ONE. (2013) 8:e62304. 10.1371/journal.pone.0062304 23638030 PMC3639274

[53] Xu J Liu Y Li Y Wang H Stewart S Van der Jeught K. Precise targeting of POLR2A as a therapeutic strategy for human triple negative breast cancer. Nat Nanotechnol. (2019) 14:388–97. 10.1038/s41565-019-0381-6 30804480 PMC6449187

[54] Mao CG Jiang SS Shen C Long T Jin H Tan QY. BCAR1 promotes proliferation and cell growth in lung adenocarcinoma via upregulation of POLR2A. Thorac Cancer. (2020) 11:3326–36. 10.1111/1759-7714.13676 33001583 PMC7606008

[55] Vincent E Villiard E Sader F Dhakal S Kwok BH Roy S. BMP signaling is essential for sustaining proximo-distal progression in regenerating axolotl limbs. Development. (2020) 147:170829. 10.1242/dev.170829 32665245

[56] Stubbs MC Min I Izzo MW Rallapalli R Derfoul A Hall DJ. The ZF87/MAZ transcription factor functions as a growth suppressor in fibroblasts. Biochem Cell Biol. (2000) 78:477–85. 10.1139/o00-053 11012087

