# Corrigendum: Identification of common Hub genes in human dermal fibroblasts stimulated by mechanical stretch at both the early and late stages

**DOI:** 10.3389/fsurg.2023.1157743

**Published:** 2023-03-15

**Authors:** Chen Dong, Wei Liu, Yu Zhang, Yajuan Song, Jing Du, Zhaosong Huang, Tong Wang, Zhou Yu, Xianjie Ma

**Affiliations:** Department of Plastic Surgery, Xijing Hospital, Fourth Military Medical University, Xi’an, China

**Keywords:** bioinformatics, genes, tissue expansion, dermis, fibroblasts, mechanical stretch

A Corrigendum on Identification of common hub genes in human dermal fibroblasts stimulated by mechanical stretch at both the early and late stages By Dong C, Liu W, Zhang Y, Song Y, Du J, Huang Z, Wang T, Yu Z and Ma X. (2022) Front. Surg. 9:846161. doi: 10.3389/fsurg.2022.846161

Due to a data processing error, there were several errors in the article because the upregulated and downregulated genes are reversed in position. Compared with the article with errors, all the “upregulation” and “downregulation” in the revised article have been reversed. A few statements in the discussion section are therefore no longer applicable and have been deleted.

Corrections have been made to section **Abstract**, subsection **Results**. This sentence previously stated:

“Seven common DEGs [DEAD-box helicase 17 (*DDX17*), exocyst complex component 7 (*EXOC7*), CASK interacting protein 1 (CASKIN1), ribonucleoprotein PTB-binding 1 (*RAVER1*), late cornified envelope 1D (*LCE1D*), *LCE1C*, and polycystin 1, transient receptor potential channel interacting (*PKD1)*] and three common DEGs [5′-3′ exoribonuclease 2 (*XRN2*), T-complex protein 1 (*TCP1*), and syntaxin 3 (*STX3*)] were shown to be upregulated and downregulated hub genes, respectively.”

The corrected sentence appears below:

Seven common DEGs [DEAD-box helicase 17 (*DDX17*), exocyst complex component 7 (*EXOC7*), CASK interacting protein 1 (CASKIN1), ribonucleoprotein PTB-binding 1 (*RAVER1*), late cornified envelope 1D (*LCE1D*), *LCE1C*, and polycystin 1, transient receptor potential channel interacting (*PKD1)*] and three common DEGs [5′-3′ exoribonuclease 2 (*XRN2*), T-complex protein 1 (*TCP1*), and syntaxin 3 (*STX3*)] were shown to be downregulated and upregulated hub genes, respectively.

A correction has been made to the section **Abstract**, subsection **Conclusions**. This sentence previously stated:

“At the early stage, there were clear changes in gene expression related to DNA and chromatin alterations; at late stages, gene expression associated with cholesterol metabolism was suppressed.”

The corrected sentence appears below:

At the early stage, there were clear changes in gene expression related to DNA and chromatin alterations; at late stages, gene expression associated with cholesterol metabolism was increased.

Corrections have been made to section **RESULTS**, subsection **Screening of DEGs**. This sentence previously stated:

“According to the selection criteria, 669 DEGs were identified at the early stage (5 h), namely, 491 upregulated and 178 downregulated genes, and 249 DEGs were identified at the late stage (24 h), namely, 149 upregulated and 100 downregulated genes.”

The corrected sentence appears below:

According to the selection criteria, 669 DEGs were identified at the early stage (5 h), namely, 491 downregulated and 178 upregulated genes, and 249 DEGs were identified at the late stage (24 h), namely, 149 downregulated and 100 upregulated genes.

Corrections have been made to section **DISCUSSION**, subsection **Early Hub Genes**. All the “upregulation” and “downregulation” in this subsection should be reversed. These sentences previously stated should be deleted:

“The simultaneous increase in histones and Ki-67 expression in HDFs under stretching indicates that active DNA packaging was involved in accelerated cell proliferation.”

“However, in this study, it was slightly downregulated [|log(FC)| = −0.511].”

“Therefore, we hypothesized that RRM2 downregulation is one of the mechanisms of Wnt/β-catenin activation.”

The corrected subsection **Early Hub Genes** appears below:

Following a 5-h cyclic mechanical stretch, representing the early stage, the top 10 hub genes identified primarily participated in DNA and chromatin alterations. Seven histone-related genes were identified and downregulated at this stage: H4 clustered histone 5 (*HIST1H4E, H4C5*), H4 clustered histone 11 (*HIST1H4J, H4C11*), H2A clustered histone 17 (*HIST1H2AM, H2AC17*), H2A clustered histone 8 (*HIST1H2AE, H2AC8*), H3 clustered histone 15 (*HIST2H3A, H3C15*), H2A clustered histone 21 (*HIST2H2AB, H2AC21*), and cluster member H1.4 linker histone (*HIST1H1E, H1-4*). Histones are fundamental structural components of chromatin (15). Eukaryotic DNA is wound around an octamer of core histones H2A, H2B, H3, and H4. The binding of the linker histone H1 promotes higher-order chromatin organization (15). The marker of proliferation Ki-67 gene (*MKI67*) encodes a nuclear protein that is required to maintain individual mitotic chromosomes dispersed in the cytoplasm following nuclear envelope disassembly and may be necessary for cell proliferation (16). DNA topoisomerase II alpha (*TOP2A*) encodes a key decatenating enzyme that alters DNA topology by binding to two double-stranded DNA molecules (17). TOP2A is generally upregulated in proliferating cells (18). However, in another study, fibroblasts, such as mouse NIH 3T3 and 3T6 cells, did not show high TOP2A expression (19). Even under certain stimuli (radiation or drugs), TOP2A expression is downregulated when fibroblasts maintain proliferation activity (20, 21). This may be related to the negative feedback regulation in fibroblasts, which prevents excessive cell proliferation (22). Another upregulated gene, ribonucleotide reductase regulatory subunit M2 (*RRM2*), encodes one of two non-identical subunits for ribonucleotide reductase, which is necessary for DNA synthesis. RRM2 also functions as a downstream factor of β-catenin as an inhibitor of Wnt signaling (23), and β-catenin activation can stimulate fibroblast proliferation (24, 25).

Corrections have been made to section **DISCUSSION**, subsection **Late Hub Genes**. These sentences previously stated should be deleted:

“All of the genes mentioned above were downregulated.”

“Ledwon et al. (4) found that, in a 24-h porcine tissue expansion model, a vast majority of DEGs were associated with metabolic processes, such as catabolism of lipids and organic acids, and were downregulated. Similarly, in our study, sterol metabolism-related DEGs were identified after a 24-h cyclic mechanical stretch and were downregulated. This demonstrates the importance of metabolic transitions in the response of HDFs to mechanical stretch in adapting and re-establishing tissue homeostasis (4).”

The corrected subsection **Late Hub Genes** appears below:

Following a 24-h cyclic mechanical stretch, representing the late stage, the top 10 hub genes identified primarily participated in cholesterol metabolism. 3-hydroxy-3-methylglutaryl-CoA synthase 1 catalyzes the condensation of acetyl-CoA with acetoacetyl-CoA to form (3S)-hydroxy-3-methylglutaryl-CoA (*HMG-CoA*), which is then converted by HMG-CoA reductase into mevalonate, a precursor for cholesterol synthesis (26). Next, mevalonate is converted into lanosterol under the action of various enzymes, namely, isopentenyl-diphosphate delta isomerase 1 and squalene epoxidase (26). Lanosterol can then be diverted into either the Bloch pathway, producing cholesterol *via* desmosterol, or the Kandutsch–Russell pathway, *via* 7-dehydrocholesterol. Methylsterol monooxygenase 1, 17-beta-hydroxysteroid dehydrogenase 7, and NAD(*P*)-dependent steroid dehydrogenase-like protein are involved in these two pathways (26). Furthermore, *INSIG1*, *STARD4*, and *C14orf1* assist in controlling sterol biosynthesis (27–29). Cholesterol is a critical regulator of lipid bilayer dynamics and plasma membrane organization in eukaryotes (30). The physical properties of the membranes depend on lipid composition; the stiffness and fluidity of the bilayers are essentially determined by the sterol content (31). Various ion channels are modulated by cellular cholesterol and partitioned into cholesterol-enriched membrane rafts (32). After cholesterol depletion, inhibition of stretch-activated cation channels is mediated *via* actin remodeling and is initiated by the disruption of lipid rafts (31). Thus, cell membrane cholesterol reduction is closely related to changes in cell morphology after stretching. Alterations in metabolic processes play a role in regulating inflammation and extracellular matrix deposition (33). In summary, changes in cholesterol metabolism are an important biological feature of HDFs under stretch conditions at the late stage and may become a potential target to help HDFs adapt more rapidly to changing environments.

Corrections have been made to the section **DISCUSSION**, subsection **Common Hub Genes**. All the “upregulation” and “downregulation” in this subsection should be reversed. The corrected subsection **Common Hub Genes** appears below:

Using overlapping DEGs between 5 and 24 h, the consistent top 10 hub genes (seven downregulated and three upregulated) were determined to be different at both stages. This shows that the biological effects of mechanical stretch varied over time. *DDX17*, *XRN2*, and *STX3* are involved in transcriptional regulation. *DDX17* encodes an important context-dependent transcriptional regulator that promotes cell growth by interacting with estrogen receptors (34). *XRN2* (*DHP1* in the yeast genus Schizosaccharomyces), an upregulated gene, triggers premature transcription termination and nucleates heterochromatin to promote meiotic gene silencing (35). *STX3*, another upregulated gene, also acts as a transcriptional regulator; inhibition of endogenous *STX3* expression alters cellular genes and promotes cell proliferation (36). *EXOC7*, *CASKIN1*, *RAVER1*, and *TCP1* are involved in cytoskeleton rearrangement. *EXOC7*, in addition to functioning in exocytosis, regulates actin at the leading edges of migrating cells, thereby coordinating cytoskeleton and membrane trafficking during cell migration (37). *CASKIN1*, a scaffold protein, regulates actin filaments (38). *RAVER1* interacts with the cytoskeletal proteins actinin and vinculin (39). *TCP1*, another upregulated gene, inhibits the transformation of fibroblasts into myofibroblasts, thus adjusting for the morphological changes caused by mechanical stretch (40). Previous studies have also reported that changes in the cytoskeleton are important results of mechanical signals and mediate the synthesis of the extracellular matrix triggered by mechanical stretch (41). Overall, hub genes related to transcriptional regulation and cytoskeleton rearrangement are potential targets for promoting HDF regeneration and alignment.

Importantly, three consistent DEGs (*LCE1D*, *LCE1C,* and *PKD1*) were downregulated and identified as hub genes. These are all closely related to skin development, according to the GO analysis. Mechanical stretch is believed to regulate intracellular calcium homeostasis, resulting in changes in a series of downstream signaling pathways (42). *LCE1D* and *LCE1C* may be downregulated in response to extracellular calcium alterations (43). However, previous studies on the function of late cornified envelope proteins were mainly conducted in keratinocytes and not in fibroblasts. Thus, the mechanisms by which *LCE1D* and *LCE1C* are involved in fibroblast differentiation and growth may be new targets for future research. *PKD1* encodes an integral membrane protein involved in the regulation of mechanotransduction signaling (44). The component of heteromeric calcium-permeable ion channels formed by *PKD1* and *PKD2* is activated by the interaction between *PKD1* and a Wnt family member, such as *WNT3A* or *WNT9B* (45). *PKD1* induces cell migration by regulating rearrangements and cell-cell mechanical adhesion (46), inhibits cell apoptosis through a PKR-eIF2α pathway (47), and regulates the cell cycle by inhibiting DNA binding (48). Therefore, downregulation of *PKD1* may be an important mechanism in HDF-sensing mechanical stretching and controlling cell growth and differentiation.

A correction has been made to section **CONCLUSION,** paragraph 1. This sentence previously stated:

“At the early stage, DNA and chromatin alterations were clearly observed; at the late stage, cholesterol metabolism was repressed to adapt to the changing environment.”

The corrected sentence appears below:

At the early stage, DNA and chromatin alterations were clearly observed; at the late stage, cholesterol metabolism was strengthened to adapt to the changing environment.

Corrections have been made to **FIGURE 3** because the upregulated and downregulated genes are reversed in position:


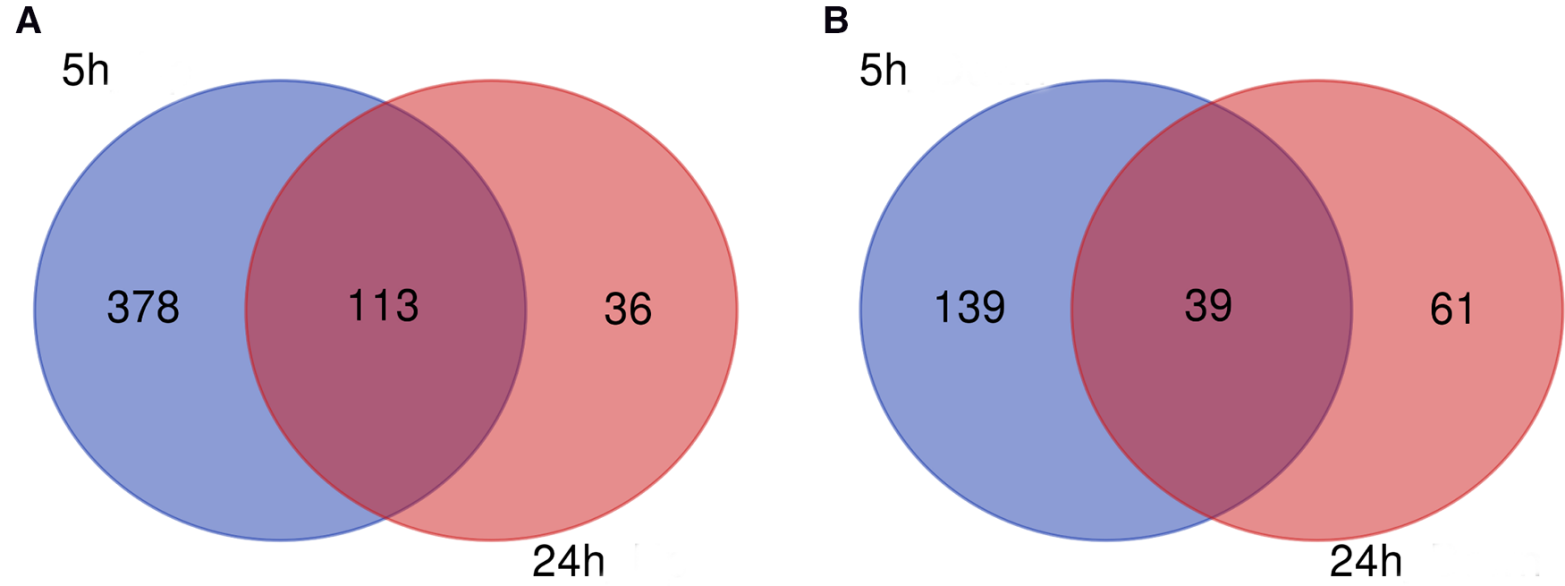


Corrections have been made to **TABLE 1** because the upregulated and downregulated genes are reversed in position:

**Table 1:** Early (5 h), later (24 h) and consistent top 10 hub Genes.

Stage	Top 10 hub genes^a^	
	Downregulated	Upregulated
Early	HIST1H4E (H4C5), HIST1H4J (H4C11), HIST1H2AM (H2AC17), HIST1H2AE (H2AC8), HIST2H3A (H3C15), HIST2H2AB (H2AC21), HIST1H1E (H1-4), MKI67	TOP2A, RRM2
Later	None	HMGCR, HMGCS1, SQLE, MSMO1, HSD17B7, IDI1, NSDHL, INSIG1, STARD4, C14orf1 (ERG28)
Consistent	DDX17, EXOC7, CASKIN1, RAVER1, LCE1D, LCE1C, PKD1	XRN2, TCP1, STX3

^a^ The genes were ranked by Maximal Clique Centrality method.

Corrections have been made to legend of **FIGURE 3** because the upregulated and downregulated genes are reversed in position:

FIGURE 3 Venn diagram. (**A**) Downregulated and (**B**) upregulated genes.

Corrections have been made to the legend of **FIGURE 5** because the upregulated and downregulated genes are reversed in position:

FIGURE 5 PPI network at 5 h. (**A**) Entire network. Downregulated and upregulated genes are represented by red and blue backgrounds, respectively. (**B**) Subnetwork for the top 10 hub genes and neighbors. The backgrounds of hub genes are colored from red to yellow by rank, and the backgrounds of neighboring genes are light blue. PPI, protein-protein interactions.

Corrections have been made to the legend of **FIGURE 6** because the upregulated and downregulated genes are reversed in position:

FIGURE 6 PPI network at 24 h. (**A**) Entire network. Downregulated and upregulated genes are represented by red and blue backgrounds, respectively. (**B**) Subnetwork for the top 10 hub genes and neighbors. The backgrounds of hub genes are colored from red to yellow by rank, and the backgrounds of neighboring genes are light blue. PPI, protein-protein interactions.

Corrections have been made to the legend of **FIGURE 7** because the upregulated and downregulated genes are reversed in position:

FIGURE 7 Common PPI network. (**A**) Entire network. Downregulated and upregulated genes are represented by red and blue backgrounds, respectively. (**B**) Subnetwork for the top 10 hub genes and neighbors. The backgrounds of hub genes are colored from red to yellow by rank, and the backgrounds of neighboring genes are light blue. PPI, protein-protein interactions. The publisher apologizes for the mistake. The original article has been updated.

The authors apologize for these error and state that these does not change the main scientific conclusions of the article in any way. The original article has been updated.

