# Supplementary material for: Identification of Common Hub Genes in Human Dermal Fibroblasts Stimulated by Mechanical Stretch at Both the Early and Late Stages
Source: Front Surg. 2022 Apr 18;9:846161. doi: 10.3389/fsurg.2022.846161 (PMC9058084; doi:10.3389/fsurg.2022.846161)
Supplement: Supplementary file 1 [file Table_1.DOCX]

| **Stage** | **Term** | **Description** | **Gene** |
| --- | --- | --- | --- |
| Early | GO:0010506 | Regulation of autophagy | ATP6V1B2, EEF1A1, MAGEA6, MEFV, NPC1, PSAP, VDAC1, BAG3, TLK2, TAB2, EXOC7, HSPB8, ATP6V1H, PHF23, FOXK1, HSPA8, HUWE1, RNF185, TAF15, AXIN2, N4BP1, SNX3 |
|  | hsa00240 | Pyrimidine metabolism | ENTPD1, DCTD, TYMP, POLR2B, RRM2, POLR3F, POLR1D, CMPK1 |
|  | GO:0035967 | Cellular response to topologically incorrect protein | HSPA8, DNAJB9, BAG3, HERPUD1, HSPB8, HERPUD2, DERL1, RNF185, DNAJA1, RRBP1, SEC23B, HYOU1, NPLOC4 |
|  | GO:0080135 | Regulation of cellular response to stress | PARP1, DNAJA1, DNAJB9, MAP2K1, PSMD10, TERC, UBE2N, AXIN2, HERPUD1, HYOU1, KAT7, TRIM32, POT1, INSIG2, ACKR3, AIDA, MAPKAP1, RNF185, DUSP15, SPRED2, ACVR1, SERPINE1, TCF7L2, KRT8, SSTR3, BAG3, DIABLO, MOAP1, ITM2C, DAG1, PPM1B, STK38, NPLOC4, TNFAIP8L1, NLRC3 |
|  | GO:0042769 | DNA damage response, detection of DNA damage | PARP1, DNAJA1, SOX4, FGF2, SERPINE1 |
|  | GO:0030855 | Epithelial cell differentiation | ACVR1, TPP1, E2F4, EVPL, FGF2, SERPINE1, PLEC, MAP2K1, PSAP, SOX4, VDAC1, PPP1R16B, HAPLN2, WDR77, CCDC78, SPRED2, ZDHHC21, LCE1C, LCE1D |
|  | GO:0006304 | DNA modification | PARP1, DNMT3A, PRMT5, CTCF, ASCC3, TREX1, TET1 |
|  | GO:0071103 | DNA conformation change | DNMT3A, H1-4, TOP2A, H4C11, H4C5, CTCF, ASCC3, TREX1, POT1, HP1BP3, TET1, H3C15, TLK2, H2AC8, H2AC17, H2AC21, INO80, MAP2K1 |
|  | GO:0046034 | ATP metabolic process | PARP1, ATP5F1C, ATP6V1B2, HSPA8, LDHA, PGAM1, SDHD, TREX1, PRKAG3, CYCS, FOXK1, ENTPD1, CMPK1, DCTD, TYMP, ACSL1, IDO1, RRM2, UAP1, GNPDA1, SLC35B3, NPC1, PRKAB1, PTGDS, MSMO1, UCP3, VDAC1, QKI, ABHD2, CYP4F8, INSIG2, GBA2 |
|  | GO:0006935 | Chemotaxis | C3AR1, DAG1, TYMP, EVX1, FGF2, SERPINE1, ENPP2, PLEC, MAP2K1, LGMN, PTAFR, CCL24, STX3, LHX3, ACKR3, IGSF9, ELMO2, JAM3, ACVR1, PKD1, SOX4, TCF7L2, LUZP1, QKI, TREX1, PPP1R16B, HIF3A |
|  | GO:0061684 | Chaperone-mediated autophagy | EEF1A1, HSPA8, BAG3 |
|  | GO:1903539 | Protein localization to postsynaptic membrane | DAG1, STX3, VAMP2, IQSEC2, CALY, JAM3, ATP6V1B2, ATP6V1H, PARP1, DNAJA1, PTPN14, TCF7L2, TCP1, PPFIA1, BAG3, HUWE1, KAT7, LDLRAP1 |
|  | GO:0048713 | Regulation of oligodendrocyte differentiation | DAG1, WDR1, PRMT5, DUSP15, MYRF, FGF2, PLEC, MAP2K1, SOX4 |
|  | GO:0022412 | Cellular process involved in reproduction in multicellular organism | ACVR1, DNMT3A, ETV6, TOP2A, QKI, ABHD2, AFF4, KNL1, WDR77, JAM3, NOBOX, ASPM, TMEM95, HOXA10, DNAJA1, SSTR3, XRN2, RNF114, HERPUD2 |
|  | GO:0031396 | Regulation of protein ubiquitination | DNAJA1, PSMD10, SOX4, UBE2N, N4BP1, HERPUD1, HUWE1, DERL1, PHF23, DNAJB9, PKD1, LGMN, ENC1, MAEA, ASCC3, TLK2, ADAMTS7, TRIM32, KCTD5, NPLOC4, RNF114, RNF185 |
|  | GO:0048024 | Regulation of mrna splicing, via spliceosome | HSPA8, SON, QKI, WTAP, DDX17, RAVER1 |
|  | GO:1901137 | Carbohydrate derivative biosynthetic process | PARP1, ATP5F1C, DCTD, ACSL1, CHST6, NPC1, RRM2, UAP1, TCF7L2, GNPDA1, TREX1, SLC35B3, CMPK1, GALNT11, MPPE1, TET1, ITM2C |
|  | GO:0072594 | Establishment of protein localization to organelle | DNAJA1, HSPA8, RN7SL1, SRP68, TCP1, BAG3, NUP93, HERPUD1, HUWE1, PITRM1, MON1B, POT1, MOAP1 |
|  | hsa05213 | Endometrial cancer | ELK1, MAP2K1, TCF7L2, AXIN2, CYCS, ACVR1, DAG1, SPRED2 |
|  | GO:0061061 | Muscle structure development | ACVR1, DAG1, KRT8, MEF2D, PLEC, TCF7L2, UTRN, PDLIM4, MSC, QKI, MAML1, WDR1, DDX17, TRIM32, SCGB3A1, FOXK1, MYMK |
| Later | GO:0016126 | Sterol biosynthetic process | HMGCR, HMGCS1, IDI1, INSIG1, MSMO1, SQLE, ERG28, NSDHL, HSD17B7, EGR1, STARD4, NPC1, CBR1, SCD |
|  | ko04721 | Synaptic vesicle cycle | ATP6V1B2, DNM1, STX3, ATP6V1H, M6PR, NEU1, NPC1, JAM3, EXOC7, TUBA3D |
|  | GO:0006900 | Vesicle budding from membrane | DNM1, INSIG1, SNX3, SEC23B |
|  | GO:0051348 | Negative regulation of transferase activity | HMGCR, PLEC, PPIA, MYCNOS, MAD2L2, POT1, CHORDC1, DVL2, EGR1, PKD1, NCK2, FAS, INSIG1 |
|  | GO:0051701 | Biological process involved in interaction with host | NPC1, PPIA, TCP1, SNX3, TNFRSF14, EXOC7 |
|  | GO:0006457 | Protein folding | PPIA, TCP1, PFDN6, HSPH1, CHORDC1, TOR2A |
|  | GO:0061061 | Muscle structure development | ACVR1, EGR1, HMGCR, MYLK, PLEC, PDLIM7, MAML1, DDX17, SCGB3A1, MYMK |
|  | GO:0043588 | Skin development | PKD1, PLEC, TXNIP, NSDHL, LCE1C, LCE1D |
|  | GO:0043433 | Negative regulation of DNA-binding transcription factor activity | PKD1, BHLHE40, MAD2L2, HES6, NLRC3, DVL2, PPIA, ACVR1, HOXA10, LHX3 |
|  | hsa05130 | Pathogenic Escherichia coli infection | KRT18, NCK2, TUBA3D, FAS, TNFRSF14 |
|  | GO:0000381 | Regulation of alternative mrna splicing, via spliceosome | WTAP, DDX17, RAVER1, AHNAK |
|  | GO:0051235 | Maintenance of location | CLIC2, INSIG1, PKD1, SQLE, FTHL17, STARD4, EGR1 |
|  | GO:0032787 | Monocarboxylic acid metabolic process | CBR1, INSIG1, ME1, NPC1, MSMO1, SCD, ME3, STARD4 |
|  | GO:0007338 | Single fertilization | HOXA10, TCP1, UBXN8, GNPDA1 |
|  | GO:0009743 | Response to carbohydrate | EGR1, HMGCR, KRT18, ME1, TXNIP |
| Common | GO:0014902 | Myotube differentiation | PLEC, MAML1, SCGB3A1, MYMK, ACVR1, DDX17 |
|  | GO:0016241 | Regulation of macroautophagy | ATP6V1B2, NPC1, EXOC7, ATP6V1H, STX3, JAM3, TUBA3D |
|  | GO:0000381 | Regulation of alternative mrna splicing, via spliceosome | WTAP, DDX17, RAVER1 |
|  | GO:0043433 | Negative regulation of DNA-binding transcription factor activity | PKD1, BHLHE40, HES6, NLRC3, ACVR1, HOXA10, LHX3 |
|  | GO:0051701 | Biological process involved in interaction with host | NPC1, TCP1, SNX3, EXOC7 |
|  | GO:0043588 | Skin development | PKD1, PLEC, LCE1C, LCE1D |
|  | GO:0007338 | Single fertilization | HOXA10, TCP1, GNPDA1 |
